# Equipping pharmacists for delivering pharmaceutical care to seniors: a qualitative systematic review of Asian seniors’ social support

**DOI:** 10.1186/s40545-023-00576-7

**Published:** 2023-06-19

**Authors:** Niken Nur Widyakusuma, Sri Suryawati, Chairun Wiedyaningsih, Probosuseno, Retna Siwi Padmawati

**Affiliations:** 1 Faculty of Medicine, Public Health, and Nursing, Universitas Gadjah Mada, Jl. Farmako Sekip Utara, Yogyakarta, 55281 Indonesia; 2 Faculty of Pharmacy, Universitas Gadjah Mada, Sekip Utara, Yogyakarta, 55281 Indonesia

**Keywords:** Social support, Seniors, Asian, Pharmaceutical care

## Abstract

**Background:**

Pharmacists must cater to seniors’ needs and communicate better in delivering pharmaceutical care. However, pharmaceutical care for seniors is unique since they commonly depend on support from their social environment. Our study aimed to collect perceptions and experiences of Asian seniors regarding social support.

**Methods:**

A qualitative systematic review of peer-reviewed articles between January 2012 and January 2022 was conducted using PubMed, Scopus, Academic Search Complete via EBSCOhost, ProQuest, and Google Scholar. Selected studies were extracted, and thematic synthesis was performed.

**Results:**

A total of 23 qualitative studies with diverse rigor were included in this review. Themes that emerged around perceptions and experiences of social support were (1) family orientation, (2) having faith in religion, (3) the importance of the elderly providing support, (4) taboos, (5) elderly self-reliance, (6) elderly fear of being a burden, and (7) differences on perceptions and experiences regarding social support. A discussion on how these results may contribute to pharmacy practice is provided.

**Conclusion:**

This study reviews the available social support for seniors and highlights its importance for pharmacists.

**Supplementary Information:**

The online version contains supplementary material available at 10.1186/s40545-023-00576-7.

## Background

The role of pharmacists in providing pharmaceutical care for seniors is rising due to an aging population worldwide. The global population aged 60 and over reached 1 billion in 2019 and was estimated to increase to 2.1 billion in 2050 [1]. Nevertheless, those aged 60 and over are generally prone to develop medication‐related problems, such as adverse drug reactions [2], poor adherence to therapy, and inappropriate drug selection [3], because of several health conditions and the use of poly-medication to control their comorbidities. This condition challenges pharmacists to provide more patient-oriented pharmaceutical services.

Providing pharmaceutical care for seniors differs from other populations. It needs more comprehension, with proficiency not only in the clinical aspects but also in the social context in which the pharmaceutical service is delivered [4, 5]. For instance, to improve treatment adherence and prevent medication-related problems, pharmacists must not only know the pharmacokinetic–pharmacodynamic changes in seniors but also notice the need for and availability of family and neighborhood support.

Social support is one of seniors' most important social determinants of health [6, 7]. It is defined as “an exchange of resources between two individuals perceived by the provider or the recipient to be intended to enhance the well-being of the recipient” [8]. The types of support are usually emotional, instrumental or tangible, informational, and appraisal [9]. The expression of emotional caring or concern, the instrumental aid, the provision of advice and guidance, and the encouragement to take opportunities are examples of each type of social support subsequently [10, 11].

It is known that seniors are in greater need of social support than adults, regarding not only receiving but also providing support [12]. Social support is important because later life is related to stressful events such as health problems, a close person’s illness or death, and loss of sources of income [13]. With declining physical and mental capacities leading to geriatric syndromes, many seniors also need informal support in medication management activities, such as obtaining medications, preparing pill boxes, assisting in medication administration, organizing and tracking medications, collecting information, and making treatment decisions [14].

However, it is also known that cultural differences play an important aspect in social support [15]. In Asia, seniors rely on their children and family members for care in old age. Multigenerational co-residence and extended family practice are also prevalent in many Asian countries [16].

Previous quantitative studies have shown that social support was essential to medication adherence in the senior population [17–20]. However, there was scarcely discussion on how seniors perceived and received social support and how understanding that support could equip pharmacists for their practice. Knowledge of social support from the perspective of Asian seniors could help pharmacists appreciate the nature of seniors’ social environment to provide pharmaceutical care that meets their needs, especially in the Asian pharmacy practice. Accordingly, we aimed to collect all available qualitative evidence and use individual qualitative data. The following research question was formulated: What themes emerged around social support from the perspective of Asian seniors?

## Methods

### Design, protocol registration, and reporting

This study was conducted as a qualitative systematic review. The protocol was registered in PROSPERO (CRD42022301602). The Preferred Reporting Items for Systematic Reviews and Meta-Analyses (PRISMA) [21] flow chart was used for the search process, and the Enhancing Transparency in Reporting the Synthesis of Qualitative Research (ENTREQ) statement [22] was used to guide a more specific reporting of a qualitative systematic review.

### Data sources and search strategy

PubMed, Scopus, Academic Search Complete via EBSCOhost, ProQuest, and Google Scholar were searched using predetermined search concepts and related terms (Table 1).

**Table 1:** Article search concepts and corresponding terms

Concept 1: Social Support	Concept 2: Perception and Experience	Concept 3: Seniors	Concept 4: Asia	Concept 5: Qualitative Data
Boolean operator: OR				
Social support, social relation*, social network*, family support, family relation*, emotional support, financial support, instrumental support, tangible support, informational support, appraisal support	Perception, experience, opinion, need, perceived, received, attitude	Aging, ageing, geriatrics, senior, older age, older adult*, elder*, older person, older people	Asia, Asian	Qualitative research, semi-structured, unstructured, in-depth, focus group, phenomenolog*, narrative, action research, case stud*, grounded, mixed-method

We define social support as emotional, instrumental, informational, and appraisal support perceived or received by the elderly [9]. We did not limit the search to the concept of pharmacist or medicine to obtain a greater possibility of social support studies (pre-planned). Therefore, the reviewers involved were medical sociologists, geriatricians, and pharmacists.

### Eligibility criteria

Full-text and peer-reviewed studies with Asian seniors as participants (60 years or older) living in Asian countries, aimed at exploring perception and experience about social support or resulting in any perception and experience regarding social support, published in English, from January 2012 to January 2022, were sought. We included any settings (community, healthcare, nursing homes) and a wide range of health conditions (physical and mental health, well-being) of the participants but excluded cognitive impairment since the condition would affect perceptions or conveying experiences of seniors. Qualitative data were defined as first-order (participants’ quotes) or second-order constructs (researcher interpretation, statements, assumptions, and ideas) [23]. Therefore, a mixed-method study would be included for the qualitative parts. We also excluded study protocols, reviews, comments, editorials, and qualitative evaluations of a social support intervention.

### Study selection and extraction

All titles and abstracts retrieved from the database searches were sent to the Mendeley reference manager. After removing duplicates, two team members altogether screened all titles and abstracts to identify studies that could meet the inclusion and exclusion criteria. The full texts of potentially eligible studies were retrieved, assessed, and extracted independently using a data extraction sheet.

### Quality assessment

The quality of the included studies was evaluated independently by two team members using the Critical Appraisal Skills Programme (CASP) appraisal tool for qualitative research [24]. Differences of opinion were resolved by consensus. We assessed the included studies for the scope and purpose, design, reflexivity, ethical consideration, analysis and interpretation, and transferability [25] to describe the rigor of the studies. We added a percentage of the + (answer yes) after each CASP 10-question to summarize the study appraisal. This summary would not be a reason to exclude the already included study.

### Data synthesis

Two team members conducted a thematic synthesis with an inductive approach that consists of three stages: the free line-by-line coding of the findings of primary studies, the organization of these free codes into related areas to construct descriptive themes, and the development of analytical themes [26]. All the texts labeled as Results or Findings in the included study were entered verbatim into QSR’s NVivo 12 Pro software for qualitative data analysis. The lines of text from a study were coded according to their meaning. The lines of text from the next studies were then coded into pre-existing or new codes. Descriptive themes were created to capture the essence of groups of initial codes. Analytical themes were then made from a group of descriptive themes to address the perceptions and experiences of social support. These stages were dependent on the judgment and insights of the reviewers. To address the heterogeneity of the included studies, we stratified the studies by settings and then identified settings of primary studies that constitute themes.

## Results

### Characteristics of the included studies and quality assessment

**Fig. 1:**
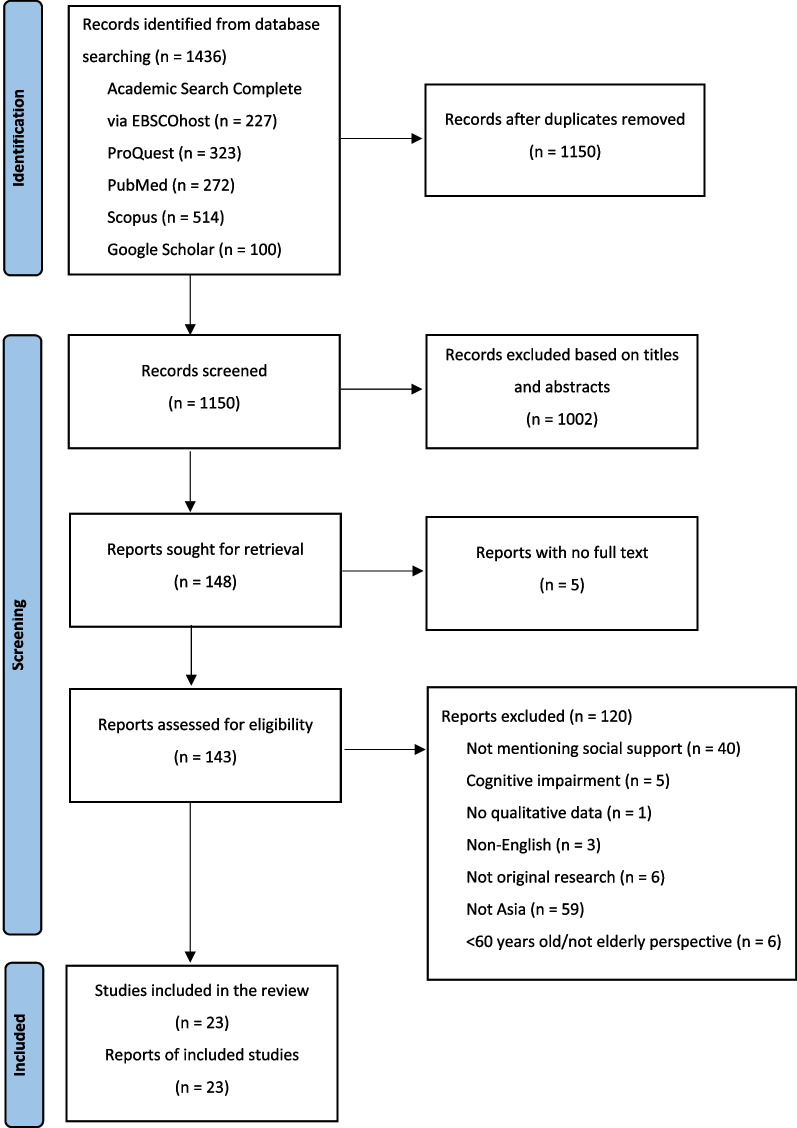
PRISMA chart

Twenty-three studies were included in this review after a search utilizing the PRISMA chart (Fig. 1). The total number of seniors who participated in the included studies was 527 individuals aged 60–94. Table 2 provides the relevant study characteristic data for the 23 included studies. The studies initially might not seek experience or perception of seniors regarding social support, but the themes obtained as the results were about support in various circumstances. Additional file 1 summarizes the quality assessment of the included studies.

**Table 2:** Characteristics of included studies (stratified by settings)

Setting	Author, year, country	Aim	Methodology*	Study results
Nursing homes	Chuang et al. 2015 [45], Taiwan	To explore the nursing home residents’ care needs	18 seniors, qualitative research, in-depth interviews, thematic analysis	Themes included Body, Economics, Environment, Mind, Preparation for death, and Social support (BEEMPS)
	Ghani et al. 2016 [49], Malaysia	To investigate social support, the forms, and the effects of social support given to seniors at the Darul Hanan Nursing Home	30 seniors, case study, in-depth interviews, thematic analysis	Themes around choosing Darul Hanan included the elderly who have no heir, elders or heirs who are living difficult lives, guaranteed security, no place to stay, easier to perform religious obligations, and neglected by family members. The forms of social support were emotional, physical, financial, and spiritual support. The effects of social support included emotional and physical effects
Shelter homes	Cassum et al. 2020 [43], Pakistan	To explore the experiences and reasons of seniors who live in shelter homes	14 seniors, descriptive exploratory design, in-depth interviews, content analysis	Themes included the circumstances of leaving home, life experiences before relocating to a shelter home, challenges to well-being before entering the care facility, coping with challenges, and the decision to live in a shelter home
Residential care homes	Lao et al., 2019 [30], China	To explore the seniors’ perceptions of family involvement in residential care homes	10 seniors, descriptive, in-depth interviews, content analysis	Themes included components of family involvement, factors influencing family involvement, impacts of family involvement on residents' lives, and promoting family involvement strategies
	Seah et al. 2020 [36], Singapore	To explore how health resources are used among seniors who are residing in senior-only households	102 seniors, descriptive, focus group discussions, thematic analysis	Themes included tapping on the internal self-care repository, maintaining and preserving informal social support, and enabling self-using environmental aids. An eco-map of aging assets was used to capture an overview of internal and external resources
Community	Almazan et al. 2019 [27], Philippines	To explore the disaster resiliency among seniors after a traumatic experience	26 seniors living alone or with family, qualitative study, focus group discussions, cross-case analysis	Themes included understanding one’s culture generates resilience that can have a huge impact in coping with disaster, holding on to their faith by praying or acting practically during adversities still promotes adaptation, gaining previous experiences is crucial to the positive outcome, and getting social support or not promotes adaptation
	Amin 2017 [28], Bangladesh	To analyze seniors’ definitions of successful aging	12 seniors living with family, grounded theory, in-depth interviews, thematic analysis	Themes included adaptation to an aging body, financial security, family and intergenerational care, and social participation
	Badriah & Sahar 2018 [39], Indonesia	To understand the experience of seniors with diabetes mellitus (DM) about their family support	8 seniors living with family, phenomenology, in-depth interviews, Colaizzi method analysis	Themes included the changes in seniors with DM, optimum family support, and suboptimal family support
	Carandang et al. 2019 [42], Philippines	To examine the perceptions of unmet needs and coping mechanisms of seniors	37 seniors, qualitative study, focus group discussions, thematic analysis	Themes included financial security, healthcare services, age-friendly environment, and family support
	Cheng et al. 2018 [44], China	To understand the intergenerational differences of social support for the seniors	30 seniors living alone or with family, qualitative research, in-depth interviews, constant comparative analysis	The young old received more formal social support and less informal social support than the older-old. The young old expected to receive more formal social support when they become the older‐old, as support from their children would be reduced due to the one‐child policy and sociocultural changes
	Han et al. 2019 [46], Singapore	To explore the impact of End Stage Renal Disease (ESRD) and dialysis and the coping strategies utilized by participants	16 ESRD seniors with family caregiver, qualitative research, in-depth interviews, framework analysis	ESRD and dialysis impacted biological/physical, psychological, and social. The strategies that participants used to cope with these biopsychosocial challenges were family support, religious/spiritual support, avoidance, and acceptance
	Harnirattisai & Vuthiarpa, 2020 [47], Thailand	To explore the perception of independent living, including the meaning, characteristics, and contributing factors of independent living	11 seniors living with family, descriptive, in-depth interviews, content analysis	Living independently meant being employed, having sufficient money for personal expenses, being capable of participating in religious activities, and having time for relaxation. The factors that contribute to independent living were personal, environment, and social support
	Kristianingrum et al. 2018 [48], Indonesia	To explore perceived family support by seniors in diabetes mellitus self-management	9 diabetic seniors living alone or with family, phenomenology, in-depth interviews, Colaizzi method analysis	The severity of family support included daily activity assistance, assistance with obtaining health services, food preparation, financial support, attention, guidance, and problem-solving. The response to family support was pleasure
	Kwan & Tam 2021 [29], Hong Kong	To examine the aging in place (AIP) experiences of seniors living in a disaster-prone rural coastal community	12 seniors living with family, case study, in-depth interviews, thematic analysis	Themes included the ability to sustain and continue seniors’ work, local community-based organizations play an instrumental role in providing social support in a disaster context, more support and resources for mitigation activities are needed, and while support exists for AIP and in a disaster situation, the seniors may not utilize such support
	Liu et al. 2015 [31], China	To explore care needs of Chinese empty-nest seniors	25 seniors living alone or with spouses, descriptive, in-depth interviews, content analysis	The care needed was home-based care. Seniors would like to have aging-in-place, home-based care by housemaid employment, or institutionalized care. However, they had some concerns about institutionalized care
	Nazari et al. 2016 [33], Iran	To investigate the meaning of perceived social support and experiences of Iranian seniors	18 seniors living alone or with family, qualitative research, in-depth interviews, content analysis	Themes included emotional, practical, informational, social companionship, providing, spiritual support, conflicts, and satisfaction with support
	Pathike et al. 2017 [34], Thailand	To explore the concept of resilience in rural Thai seniors	35 seniors living alone or with family, ethnography, semi-structured interviews and observation, thematic analysis	The main theme was moving on. The sub-themes included keeping a job and earning a living, having Jai-Yai to fight for life, accepting a situation (Plong and Taam-Jai), expressing difficulty, and connecting with people, beliefs, and customs
	Rittirong et al. 2014 [35], Thailand	To explore rural seniors’ preferences for support	102 seniors, qualitative study, focus group discussions, thematic analysis	The types of support were meal preparation, personal care, transportation, and financial and emotional support. Male and female seniors’ preferences were slightly different for genderized tasks. Social closeness and geographical proximity mattered
	Shiraz et al. 2020 [37], Singapore	To examine seniors’ perceptions of physical, psychological, and social health and the processes of adaptation and self-management	40 seniors, exploratory, in-depth interviews, thematic analysis	Themes around physical, psychological, and social health perceptions included slowing down, relationship harmony, financial harmony, social connectedness, and eating together. Themes around adaptation and self-management included keep moving, keep learning, adopting avoidant coping behaviors, “It feels good to do good,” “Power of Prayer,” and social participation
	Sta Maria et al. 2018 [32], Philippines	To explore the quality of social relationships of senior Filipino church members	6 seniors living with family, qualitative research, in-depth interviews, thematic analysis	Themes included forms of support and nonsupport
	Tabari et al. 2017 [38], Iran	To identify factors that affect the mental health of seniors	15 seniors living alone or with family, qualitative research, in-depth interviews, content analysis	Themes included interaction and worthiness. Main categories included communication/relationship, empathy/compassion, entertainment/amusement, support, and respect
	Tsuji & Khan 2016 [40], Japan	To investigate the relationship between social support and the life satisfaction of seniors in Japan	5 seniors living alone or with family, qualitative research, in-depth interviews, interpretative phenomenological analysis	Themes included gender difference, transition of role from carer to being cared for, and reciprocity
	Yoo 2013 [41], Korea	To explore how seniors perceive and desire social support in an aging society	21 seniors living alone or with family, qualitative research, in-depth interviews, thematic analysis	Themes included no or denied support, not being greedy and shameless, and justification and hopelessness

*Some studies did not provide information on living arrangements of the participants

### Perceptions and experiences of Asian seniors regarding social support

We identified seven analytical themes on how Asian seniors perceive and experience social support: (1) family orientation, (2) having faith in religion, (3) the importance of the elderly providing support, (4) taboos, (5) elderly self-reliance, (6) elderly fear of being a burden, and (7) differences on perceptions and experiences regarding social support. The first analytical theme, “family orientation,” was prominent since 22 out of 23 studies showed experiences and perceptions of support for family [27–48]. This analytical theme was built upon many descriptive themes and codes which showed that family almost always be the directions or underlying view of mind. The second analytical theme, “having faith in religion,” was also prominent since 17 of 23 articles showed experiences and perceptions of support for seniors’ religious faith [27, 28, 32–39, 41–43, 45–47, 49]. Table 3 provides the themes, examples of quotations, and settings of primary studies that constitute themes.

**Table 3:** Themes, example of quotations, and settings of primary studies that constitute themes

Analytical themes	Descriptive themes	Example of quotations	Settings of primary studies that constitute themes
Family orientation	Expectation for offspring	“… I do not need their money. All I needed is their affection and emotional support. Everyone around talks about this. It is so shameful that my own children do not visit me or care for me.” (65-year-old female, rural, literate) [28]	All settings (nursing homes, residential care homes, shelter homes, community) [28–48]
	Experiencing disrespect from family members	Our 79-year-old female participant shared, “I am also forgetful, sometimes she gets irritated, and she tells me, ‘You said that already, you keep repeating yourself’” (translated from Filipino). This quote expresses experiences of disrespect when some social network members do not regard the elderly positively when symptoms of aging, such as forgetfulness, are exhibited [32]	Shelter homes, community [32, 33, 43]
	Appreciation on family reputation	Influenced by a traditional mindset, some seniors feel that it would harm their children’s dignity and their family's reputation if they were to reside in a nursing home for supportive care: “If I went to a nursing home in the future, then people would think that my children were impious, and my children might feel their reputation was impaired.” [31]	Community [28, 31, 33, 37, 41]
	Gathering and celebrating festive days with family and friends	However, most (*n* = 7) of them mention that the visits are often during major festivals, as a participant explained, “Yes! I have eight or nine great-grandchildren. But I do not see them much. Well, yes, they do come to see me. During the big festival, these children come to visit me to have a festival gathering dinner. Some of them would drive all the way back here to visit.” [29]	Community [29, 38]
	Food as a symbol of family cares	The extra physiological support for food, clothing and other living necessities from family members who were present was a symbol of “family cares” that they live better lives at the homes: “My daughter bought me some snacks as dinner was served at 5 pm and I ate little. I was hungry by midnight.” [30]	Nursing homes, residential care homes, community [30, 34, 37, 45, 49]
Having faith in religion	Expectation for spiritual support	Stronger faith was much more important, especially if a life-threatening situation comes in their way: “This is the time that we should look up to God and pray. We can surpass this.” [27]	Nursing homes, shelter homes, community [27, 28, 32–35, 37–39, 41, 43, 45, 46, 49]
	Accepting situations	“Even though health control, medication, and diet are so important, but more important is surrender to God, accepting destiny happened and sincere” [39]	Community [34, 39, 46]
	Religious social support	“Religious affiliation and practice was frequently reported in our sample, with religious affiliations ranging from Christianity, Buddhism, Taoism, Muslim, and Hinduism. Despite religious affiliation, older adults identified the positive impact engaging in religious activities had on their social health. They highlighted how being part of a religious group allowed them to be around people who shared similar beliefs, and they felt a sense of belonging and purpose” [37]	Residential care homes, community [35–37, 41, 42, 46, 47]
The importance of elderly providing support		“Helping and sharing significant concern for others allowed some older adults to feel better about themselves. It also gave many the motivation to look after their own health, especially if they were providing care to someone close to them, e.g., family member, partner, or close friend” [37]	Community [28, 32, 33, 35, 37, 39, 44]
Taboos		“I have told my daughter (about my death) and wanted to say a nice goodbye. She does not want to listen to me. She becomes angry when I mention it. I want to talk, but she does not want to listen” (Mr. Hsu). However, discussing death is somewhat of a taboo in Chinese society [45]	Shelter homes, nursing homes, community [28, 37, 41, 43, 45]
Elderly self-reliance		“The young‐old reported that there was a lack of formal instrumental support in the community they live. They mainly do the housekeeping work by themselves, as most of them are independent at this stage” [44]	Nursing homes, residential care homes, community [29, 31, 34, 36, 42, 44, 45, 47]
Elderly fear of being a burden		“My legs hurts when I walk or try to climb stairs. So far, I can take care of myself. I hope I would go (die) before I get bedridden” [28]	Shelter homes, residential care homes, community [28, 30, 35, 42, 43, 46, 47]
Differences on perceptions and experiences regarding social support	Younger-old vs. Older-old	“The young‐old reported that the formal emotional support increased compared to their parents’ generation as the community increasingly organizes social activities. The informal emotional support from their children and neighbors, however, decreased compared to their parents' generation and the older‐old living in the same community. The neighbors were all Beijing local residents in the past. We understood each other and cared about each other. The neighbors were like families. If my neighbor’s relatives came and there was no place to stay, they just came to stay in my home. Now is different. People become selfish, especially these migrants. The society has changed a lot” [44]	Residential care homes, community [36, 44]
	Male vs. female	“At first, men expressed social support, which was more related to formal, including social role and local meeting. In addition to this, their expression of life events which related to life satisfaction in the past and future was their business and social role. Compared to males, females discoursed their social support and life satisfaction as being related to friends and family. Additionally, in terms of life satisfaction in the past, current, and future, females’ life events related more to family events and situation” [40]	Community [28, 35, 40, 41]
	Having family vs. having no family	However, this is contrary to groups of older adults without families. Looking for alternative sources of food and shelter is much more important than praying. Their life is focused on action and not solely prayer: “I need to look for alternative resources. If I will rely on asking for a miracle, I might be dead.” [27]	Community [27]

Although Asian seniors were thought to rely on family, some included studies provided the perspectives of seniors who have no family or live without family. They tend to act practically than rely on praying [27] or choose nursing homes as their “homes” [49]. However, younger seniors, in good physical condition and capable of self-care, prefer home-based care rather than institutionalized care. Those who find it difficult to live alone intend to receive supportive care from society [31].

Most included studies also discussed faith or spiritual support. Many Asian seniors have a deep faith in religion which is a significant source of strength when facing life stressors [43]. However, although social support generally assists seniors positively, sometimes that support provides a negative experience, such as disrespect [32, 33, 43] or feeling of being a burden [43, 46, 47].

## Discussions

Understanding how family and religious faith support (or unsupported) seniors might sensitize pharmacists to psychological and sociological factors that might be subsequently involved in medication taking and health-related behaviors. It is known that patient survival is improved when social and emotional factors are explicitly considered by healthcare providers [50]. Moreover, understanding social support would improve pharmacist communication, which previously noted as ineffective two-way communication between pharmacist and patient [51]. Since the nonadherence problems are located within the inefficient communication process or in the lack of rapport with patients [52], improving communication might improve patient adherence.

Assessing whether seniors have appropriate family support is the most important since family or relatives will assist seniors in adhering to and benefiting from treatment recommendations. Some studies indicated pharmacists should understand that family members are essential to patients’ medication [33, 48]. A study in Thailand also revealed that elderly individuals with a daughter as a caretaker were approximately eight times more likely to adhere to their antihypertensive treatment than patients with no caretaker [53].

However, there is an increasing trend of seniors living alone in Asia, such as Japan, South Korea, and Taiwan [54]. Nevertheless, living alone does not necessarily mean no family support because nonresident family members can still provide support [55]. For seniors living in households separate from family members, pharmacists might help these seniors maintain their independence. Pharmacists should be aware that seniors expect to be self-reliant and not to be a burden to anyone.

Religious or spiritual support might correspond with medication use and adherence. Pharmacists may encounter scenarios and circumstances where communication about faith becomes necessary, such as discussing chronic disease management in religious communities. Because believing in a higher power enables seniors to face difficult times with an optimistic and resilient attitude [37], pharmacists should not go against this faith. Instead, encouraging seniors to develop self-reflexivity through communication might promote better adherence.

Other things to be considered are that receiving and providing support is also crucial for seniors [37]. Being involved and active in any community, such as a church community [45], would make seniors feel useful as they can provide emotional support and friendship to others, share information and encouragement [32], and remain active as long as possible. A previous study even proved that providing social support for elders is more important than receiving it [12]. To address this need, there are usually ranges of communities a pharmacist can suggest to seniors, from neighborhood-based to hospital-based communities.

Pharmacists must understand taboos and other sensitive issues around seniors’ conditions. Discussing death with seniors might be considered taboo [45], but pharmacists involved in end-of-life and palliative care would eventually encounter this conversation with seniors. Pharmacists must also know that discussing seniors’ needs would be challenging as imposing needs and problems on others might also be considered inappropriate, as the included studies indicated [37, 41].

However, seniors’ perceptions and experiences regarding social support may vary across regions and circumstances. The included studies indicated that younger-old, male, or living with a family will have different needs or perspectives than older-old, female, or having no family. Tailoring the health education message to the needs of seniors would be more helpful. Patients take information and process it within their cognitive framework based on their interpretation of their own experiences [4]. Thus, even seniors would act differently and selectively based on their needs and circumstances.

### Implication for pharmacy practice

Improving pharmacist communication with seniors is the central recommendation from this review. The authors suggest points that might be incorporated into a standard procedure of pharmacist communication with seniors, such as ensuring the availability and ability of social support as well as assessing faith and beliefs related to medicine or health behavior. Since it is common in Asian culture to greet and ask about the condition of somebody’s family and relatives, it might be easier for pharmacists to do this communication. It will give insight into how pharmacists may involve any support available for individual seniors. For example, for independent seniors, it might be appropriate to ensure that their medication self-management at home is correct and to encourage them to be active in the community; for more dependent seniors who live with family members, it might be appropriate to educate through their family; or for seniors who live without family, it might be appropriate to ensure the availability of nearby relatives or neighbors to take them to regular check-ups, and so on.

### Strength and limitations

From the pharmacy perspective, this review gives insights into pharmacist communication approach to elderly patients. Nevertheless, this review had some limitations. First, as a qualitative synthesis, data retrieved were thrice removed, which means we interpreted the experiences and perceptions that the original researchers interpreted from the interpretation of the seniors themselves as study participants [56]. We were not in the place and context when the primary data were collected, but rather we discussed the data in a quite diverse expertise background of reviewers. Second, we only included articles in English; there might be other comprehensive studies from Asia using non-English languages. Third, the search strategy in this review was not directly associated with pharmacy or medicine, but our discussion attempts to link the available evidence about social support with pharmacy practice.

## Conclusions

The present review affirmed the evidence from Asian seniors that they are more affected by family support and religious faith. Pharmacists should incorporate family and religious faith approaches in communication with seniors for an effective pharmaceutical service.

### Supplementary Information

**Additional file 1.** The summary of quality assessment of the included studies.

**Additional file 2.** ENTREQ Statement.

## Data Availability

All data for analysis in this review is in the public domain.

## Abbreviations

PRISMA: Preferred Reporting Items for Systematic Reviews and Meta-Analyses
ENTREQ: Enhancing Transparency in Reporting the Synthesis of Qualitative Research
CASP: Critical Appraisal Skills Programme
