# Supplementary material for: Equipping pharmacists for delivering pharmaceutical care to seniors: a qualitative systematic review of Asian seniors’ social support
Source: J Pharm Policy Pract. 2023 Jun 19;16:75. doi: 10.1186/s40545-023-00576-7 (PMC10278261; doi:10.1186/s40545-023-00576-7)
Supplement: Supplementary file 1 — Additional file 1. The summary of quality assessment of the included studies. [file 40545_2023_576_MOESM1_ESM.docx]

Additional File 1. Quality Assessment of Included Studies

| **No.** | **Item** | **Almazan et al., 2019** | **Amin, 2017** | **Badriah & Sahar, 2018** | **Carandang et al., 2019** | **Cassum et al., 2020** | **Cheng et al., 2018** | **Chuang et al., 2015** | **Ghani et al., 2016** | **Han et al., 2019** | **Harnirattisai & Vuthiarpa, 2020** | **Kristianingrum et al., 2018** | **Kwan & Tam, 2021** | **Lao et al., 2019** | **Liu et al., 2015** | **Nazari et al., 2016** | **Pathike et al., 2017** | **Rittirong et al., 2014** | **Seah et al., 2020** | **Shiraz et al., 2020** | **Sta Maria et al., 2018** | **Tabari et al., 2017** | **Tsuji & Khan, 2016** | **Y00, 2013** |
| --- | --- | --- | --- | --- | --- | --- | --- | --- | --- | --- | --- | --- | --- | --- | --- | --- | --- | --- | --- | --- | --- | --- | --- | --- |
| 1 | Were there a clear statement of the research aims? | + | + | + | + | + | + | + | + | + | + | + | + | + | + | + | + | + | + | + | + | + | + | + |
| 2 | Is a qualitative methodology appropriate? | + | + | + | + | + | + | + | + | + | + | + | + | + | + | + | + | + | + | + | + | + | + | + |
| 3 | Was the research design appropriate to address the aims of the research? | + | + | + | + | + | + | + | + | + | + | + | + | + | + | + | + | + | + | + | + | + | + | + |
| 4 | Was the recruitment strategy appropriate to the aims of the research? | ? | + | + | + | ? | + | + | ? | + | + | ? | + | + | + | ? | + | + | + | + | + | + | ? | + |
| 5 | Was the data collected in a way that addressed the research issue? | + | + | ? | + | + | + | + | ? | ? | + | + | + | + | + | + | + | + | + | + | + | + | ? | + |
| 6 | Has the relationship between researcher and participants been adequately considered? | + | - | - | - | - | - | - | - | - | - | - | - | - | - | - | - | - | - | - | - | - | - | - |
| 7 | Have ethical issues been taken into consideration? | + | + | + | + | + | + | + | ? | + | + | + | + | + | + | + | + | ? | + | + | ? | + | + | ? |
| 8 | Was the data analysis sufficiently rigorous? | + | - | - | + | + | + | - | - | + | + | + | + | + | + | + | + | + | + | + | + | + | + | - |
| 9 | Is there a clear statement of findings? | + | + | + | + | + | + | + | + | + | + | + | + | + | + | + | + | + | + | + | + | + | + | + |
| 10 | Will the results help locally? | + | + | ? | + | + | + | + | ? | + | + | ? | + | + | + | + | + | + | + | + | + | + | + | + |
|  | % Score of + | 90 | 80 | 60 | 90 | 80 | 90 | 80 | 40 | 80 | 90 | 70 | 90 | 90 | 90 | 80 | 90 | 80 | 90 | 90 | 80 | 90 | 70 | 70 |
